# Beyond city expansion: multi-scale environmental impacts of urban megaregion formation in China

**DOI:** 10.1093/nsr/nwab107

**Published:** 2021-06-22

**Authors:** Weiqi Zhou, Wenjuan Yu, Yuguo Qian, Lijian Han, Steward T A Pickett, Jing Wang, Weifeng Li, Zhiyun Ouyang

**Affiliations:** State Key Laboratory of Urban and Regional Ecology, Research Center for Eco-Environmental Sciences, Chinese Academy of Sciences, Beijing 100085, China; College of Resources and Environment, University of Chinese Academy of Sciences, Beijing 100049, China; Beijing Urban Ecosystem Research Station, Research Center for Eco-Environmental Sciences, Chinese Academy of Sciences, Beijing 100085, China; State Key Laboratory of Urban and Regional Ecology, Research Center for Eco-Environmental Sciences, Chinese Academy of Sciences, Beijing 100085, China; State Key Laboratory of Urban and Regional Ecology, Research Center for Eco-Environmental Sciences, Chinese Academy of Sciences, Beijing 100085, China; State Key Laboratory of Urban and Regional Ecology, Research Center for Eco-Environmental Sciences, Chinese Academy of Sciences, Beijing 100085, China; Cary Institute of Ecosystem Studies, Millbrook, NY 12545, USA; State Key Laboratory of Urban and Regional Ecology, Research Center for Eco-Environmental Sciences, Chinese Academy of Sciences, Beijing 100085, China; Beijing Urban Ecosystem Research Station, Research Center for Eco-Environmental Sciences, Chinese Academy of Sciences, Beijing 100085, China; State Key Laboratory of Urban and Regional Ecology, Research Center for Eco-Environmental Sciences, Chinese Academy of Sciences, Beijing 100085, China; State Key Laboratory of Urban and Regional Ecology, Research Center for Eco-Environmental Sciences, Chinese Academy of Sciences, Beijing 100085, China; College of Resources and Environment, University of Chinese Academy of Sciences, Beijing 100049, China

**Keywords:** urbanization, urban and regional sustainability, landscape change, air pollution, urban heat island

## Abstract

Environmental degradation caused by rapid urbanization is a pressing global issue. However, little is known about how urban changes operate and affect environments across multiple scales. Focusing on China, we found urbanization was indeed massive from 2000 to 2015, but it was also very uneven, exhibiting high internal city dynamics. Urban areas in China as a whole became less green, warmer, and had exacerbated PM_2.5_ pollution. However, environmental impacts differed in newly developed versus older areas of cities. Adverse impacts were prominent in newly urbanized areas, while old urban areas generally showed improved environmental quality. In addition, regional environmental issues are emerging as cities expand, connect and interact to form urban megaregions. To turn urbanization into an opportunity for, rather than an obstacle to, sustainable development, we must move beyond documenting urban expansion to understand the environmental consequences of both internal city dynamics and the formation of urban megaregions.

## Teaser

Massive urbanization in China caused overall environmental degradation: environmental quality in old urban areas was generally improved but adverse impacts were prominent in newly urbanized areas.

## INTRODUCTION

Rapid urbanization is one of the most pressing issues confronting the world, and nowhere is that spread and intensification more massive than in China. Urbanization has long been considered a major driver of many ecological and environmental problems [1]. Urbanization converts farmland, forests, wetland, grasslands and deserts to built-up areas as old cities expand and new cities are established. Numerous studies have documented the adverse ecological and environmental impacts of this urban expansion and its associated land conversion from local to global scales [1–3]. Not only is urbanization a major force of changes in land use and land cover worldwide [4], directly driving the loss of arable land and wildland habitat [3,5], but also it increases habitat fragmentation and threatens biodiversity [2,6,7], contributes to urban and regional warming [8,9], increases surface runoff and causes water pollution [10], and results in high concentrations of air pollutants [11,12]. However, much less is known about two additional important facets of urban spatial change—the formation of urban megaregions as cities in a region expand and interlink, and the internal dynamics in existing cities.

Urban megaregions are an increasingly important spatial form throughout the world [13–16]. For example, urban megaregions, which are sometimes referred to as urban agglomerations, have been recognized as the major urban form for future urbanization in the ‘National New-Type of Urbanization Plan’ of China [17,18]. Even in long urbanized areas such as the United States, urban megaregions are of growing importance [19]. Existing cities, expanding suburbs, new urban settlements and new infrastructure are knitting together into megaregions. Although the literatures on urban planning and design [20], and on economic globalization recognize the significance of regional connectivity of urban form [21], the spatial processes and their ecological impacts are less well known. Also under investigated is a second kind of change—the internal dynamics of existing cities [22]. Internal changes include, for example, the demolition and replacement of old buildings and infrastructure with new versions, the creation of a temporary land bank during replacement, or the infill of ‘left over’ land previously unbuilt. Such changes may have profound ecological impacts, but also provide enormous opportunities to introduce sustainable technologies and practices at many scales [23,24].

How do the emergence of important characteristics at the megaregional scale and the persistence of internal city dynamics affect ecosystems and the environment? This paper examines the multi-scaled patterns of rapid urban change in China and their impacts on three important urban effects: (1) the stock of arable land and habitat, including the vegetation component of urban systems, (2) land surface temperature as a driver of heat island effects and (3) the concentration of particulate air pollution (PM_2.5_). These variables are important to human health and wellbeing, to biological diversity, and to design and planning decisions at multiple scales in urban systems. Understanding the multi-scaled patterns of rapid urban change and associated ecological impacts has implications for urban sustainability, regional connectivities, conservation and non-urban land uses. Although social characteristics of megaregion formation and internal urban dynamics are undoubtedly important, the scope of this paper is the spatial changes and key environmental aspects of those changes.

## RESULTS

### Spatial and temporal patterns of urban expansion in China

China experienced rapid and massive, but uneven urban expansion from 2000 to 2015. Developed land in the mainland of China increased by approximately 80 000 km^2^ from 211 756 km^2^ (or about 2.2%) in 2000 to 291 747 km^2^ (about 3.0%) in 2015, with an annual growth rate of 2.5%. Many cities experienced rapid and dramatic urban expansion. For example, Shenzhen, a fishing town in the 1970s, grew to a megacity with more than 20 million people in less than 40 years, with its urban land area expanding from 26.5 km^2^ in 1980 to 946.1 km^2^ in 2017. The magnitude of developed land in some cities, such as Wuhan, Hefei and Haikou, almost doubled in size. In contrast, in western China, although cities such as Chengdu and Guiyang expanded, they fell far short of doubling from 2000 to 2015 (Supplementary Table 1).

**Figure 1.**
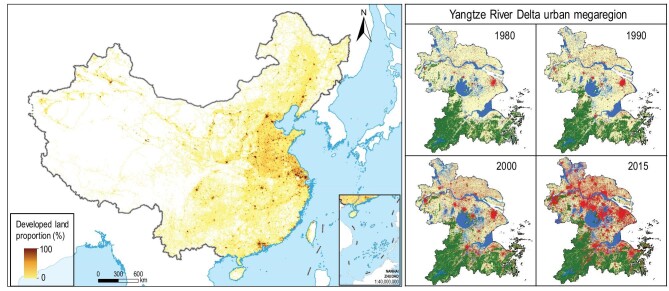
Spatial pattern of developed land in China in 2015 (left panel), and the emergence of urban megaregions from 1980 to 2015 using the Yangtze River Delta as an example (right panel). The land cover maps of the Yangtze River Delta region from 1980 to 2015 show a dramatic urban expansion and the emergence of an urban megaregion. Red color in the land cover maps represents urban area; dark green, blue and yellow represent forest, water and farmland, respectively.

While all cities experienced urban growth, urban expansion was mostly concentrated in a few regions, showing remarkable regional variation (Fig. 1; Supplementary Table 1). The newly developed land was mostly concentrated in the eastern and central regions. The cover of developed land in the eastern region increased from 8.6% in 2000 to 12.4% in 2015, growing at a rate of 43.4%; whereas that in the western region only increased from 0.6% to 1.0% (Supplementary Table 1). Urban expansion in existing or planned urban megaregions also varied greatly (Supplementary Fig. 1). For example, the proportional cover of developed land in the Yangtze River Delta, one of the most developed urban megaregions, increased from 4.1% in 1980 to 24.8% in 2015, an increase of 21 376 km^2^, which is approximately half the size of Demark. In contrast, the proportional cover of developed land in the ChengYu (Chengdu-Chongqing) urban megaregion was only 3.4%, or slightly higher than the national average (3.0%) in 2015. In addition, there are large variations in the magnitude and speed of urban expansion among cities (Supplementary Table 1).

The concentration of urban expansion in certain regions has resulted in the emergence of urban megaregions (Fig. 1). Existing cities, expanding suburbs and new urban settlements, and infrastructures have been gradually knitting into urban megaregions. These megaregions are concentrations of urban population and economic activities, with high proportional cover of developed land, and good connections by high-speed trains and express highways. Examples are the Yangtze River Delta megaregion, the Pearl River Delta megaregion and the Beijing-Tianjin-Hebei megaregion (Supplementary Fig. 1). The Yangtze River Delta urban megaregion, for example, covers an area of 115 626 km^2^ (1.2% of the whole nation), but has a total population of 80.2 million (5.8%), and contributed to 12.3% of the national GDP circa 2015.

In addition to urban expansion, internal dynamics in Chinese cities are high. Results from the analysis of nine major cities in China using very high spatial resolution data show that land cover such as greenspace in urban core areas has changed dramatically (Supplementary Fig. 2C). For all nine cities, within-city greenspace dynamics were high, with the magnitude of greenspace loss ranging from 41.9 km^2^ to 72.3 km^2^ in 2005–2010, while also showing great gains of newly established greenspace during the same time period (Supplementary Fig. 2C). For example, the area within the fifth ring road of Beijing added 70.1 km^2^ (10.5% of the total area) of new greenspace, while 42.0 km^2^ (6.3%) were converted to impervious surfaces in the period from 2005 to 2010. Similarly, within the outer ring road area of Shanghai, the most well-developed part of the city, more than 82 km^2^ (up to 10% of the total land area) were under construction or waiting for development in 2010. Some such changes occurred in the form of large patches, but most were small in size (Supplementary Fig. 2B).

### Impacts of urban expansion on land conversion

Urban expansion was a major driver of land conversion in China. From 2000 to 2015, farmlands were the most affected by urban expansion, followed by grassland and forest lands (Supplementary Fig. 3). Among the approximately 80 000 km^2^ land converted to urban, 70.9% was farmland, 11.1% was grassland, 10.1% was forest and 4.2% was wetland. Urban expansion was the dominant driver of farmland loss, accounting for 43.2% of the total loss at the national scale (Supplementary Table 2). The contributions of urban expansion to farmland loss were more prominent in urban megaregions than around isolated cities (Supplementary Table 2). For example, in the Pearl River Delta, Wuhan and Chang-Zhu-Tan megaregions, more than 70% of the newly urbanized areas were converted from farmland. From 2000 to 2015, an increasing proportion of developed land was converted from wetlands (Supplementary Fig. 3B). In addition to the loss of farmland and natural land, urbanization is a major driver of land fragmentation in China. For example, farmland increasingly became fragmented with urban expansion, as indicated by the declining mean patch size of farmland (Supplementary Fig. 4). Similarly, urban expansion resulted in forest fragmentation; however, the net pattern became less fragmented because of ecological forest restoration [7].

### Impacts of urban spatial change on EVI

**Figure 2.**
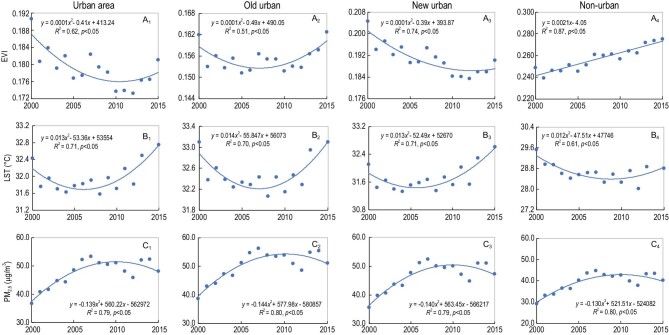
Trends of EVI, LST and PM_2.5_ in urban, old urban, new urban and non-urban areas. The old urban area refers to the land in a prefectural city that was developed before 2000, while the new urban area was the land that was developed from 2000 to 2015. Urban includes both the new and old. The panels show the aggregated mean of urban, old urban, new urban and non-urban for 305 cities and their changes from 2000 to 2015, respectively. Specifically, panels (A_1_) to (A_4_) show the trends of EVI for urban, old urban, new urban and non-urban areas from 2000 to 2015, (B_1_) to (B_4_) show the trends of LST, and (C_1_) to (C_4_) show the trends of PM_2.5_ concentration.

From 2000 to 2015, urban areas in China generally became less green, as indicated by the trend of reduced enhanced vegetation index (EVI; Fig. 2). But from 2012, there was a trend of turning green (Fig. 2A_1_). The dynamics of EVI differed between older urban areas that had developed before 2000 and urban areas that developed after that date. The decrease in urban EVI was mainly a result of patterns in new urban areas, which is likely to have resulted from loss of farmland and natural land during urban expansion. In contrast, the old urban areas had a U-shape trend of change in EVI. EVI first decreased in old urban areas, but has increased since 2008 (Fig. 2A_2_).

**Figure 3.**
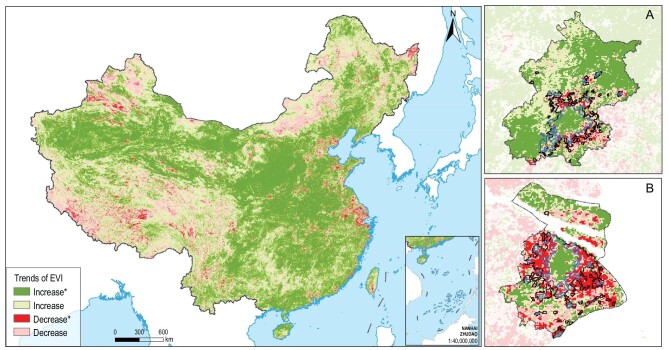
Spatial pattern of EVI trends in 2000–2015 for China and two major cities. * Coefficient is significant at the 0.05 level. (A) and (B) are the cities of Beijing and Shanghai, located in the eastern coastal area. The blue line represents the boundary of old urban areas, the black line represents the boundary of new urban areas and the dotted line represents the administrative boundary.

Urban expansion had significant adverse impact on EVI. From 2000 to 2015, 66.3% of the land in newly urbanized areas had reduced EVI, with 42.9% of cases being significant. In contrast, in non-urban areas, only 18.1% of the land had reduced EVI, with 2.7% being significant. Urban areas as a whole had 44.0% of land with increased EVI, and 56.0% with reduced EVI, in contrast to 81.6% of land nationwide with increased EVI (Fig. 3 and Supplementary Table 3). The lands with significant trends of EVI reduction were mostly located in the eastern region where the largest urban expansion occurred (Fig. 3).

### Impacts of urban spatial change on local climate

Urban areas in general become warmer from 2000 to 2015, as indicated by the trend of changes in land surface temperature (LST; Fig. 2B_1_). The averaged LST decreased slightly from 2000 to 2004, but then increased continually, forming a U-shape curve. Both the new and old urban areas had a U-shape change in LST, and have become warmer in recent years. However, the warming was more prominent in new urban areas, showing the expected adverse impacts of urban expansion on local and regional climate (Fig. 2B_2_ and B_3_).

**Figure 4.**
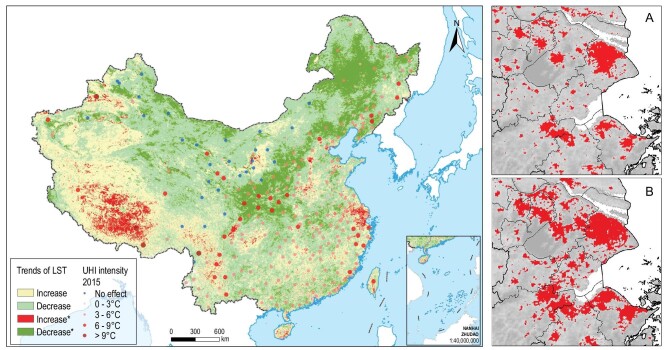
LST trends and urban heat island intensity in China from 2000 to 2015. * Coefficient is significant at the 0.05 level. Figure on the left shows that a large proportion of land (66.9%) had a trend of LST decrease from 2000 to 2015 (in green). But the majority of cities had UHI intensity greater than 3.0°C. (A) and (B) show locations with higher temperatures (red color) in the Yangtze River Delta megaregion in 2000 and 2015, respectively. The isolated heat islands in 2000 expanded and connected to one another, forming the heat ‘archipelagos’ in 2015.

From 2000 to 2015, 60.1% of the urban areas had increasing LST, among which 17.9% had significant increases. In contrast, nationwide only 33.1% of the land had increasing LST (Fig. 4), among which only 3.6% had significant increases (Supplementary Table 4). Although urban areas account for only a small proportion of the nation's land, the 17.9% with significant LST increase occurred in urban areas. The increase of LST in urban areas coupled with decrease of LST in non-urban areas resulted in intensified urban heat island (UHI) effects, a phenomenon of higher land and/or air temperatures in urban areas than in their surrounding regions. UHI is one of the most widely recognized impacts of urban expansion [25]. In 2000, 84.5% of Chinese cities showed UHI effects, but by 2015 this proportion increased to 90.9%, when more than 70.0% of the cities had temperatures greater than 3.0°C warmer than their surrounding non-urban regions (Supplementary Fig. 5). Not only did the number of cities with UHI increase, but also the intensity (i.e. the magnitude of temperature differences) of  UHI increased. More than two-thirds of the cities had intensified UHI (Supplementary Fig. 5). In particular, the number of cities with temperature differences greater than 3.0°C and 6.0°C increased by 31 and 10, respectively. In contrast, the number of cities without noticeable UHI declined by 22 and the number of cities with temperature differences less than 3.0°C declined by 19.

**Figure 5.**
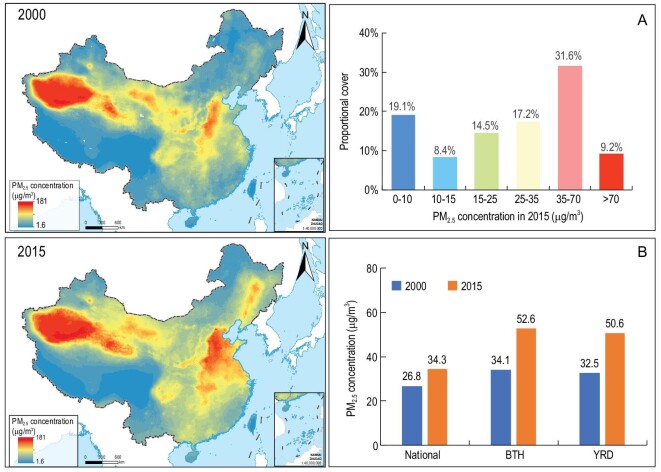
Spatial pattern of PM_2.5_ concentration in 2000 and 2015. Figures on the left show the spatial patterns of PM_2.5_ concentration in 2000 and 2015. (A) is the proportional cover of each concentration class based on the interim target released by the World Health Organization (WHO). (B) shows the averaged PM_2.5_ concentration in the Beijing-Tianjin-Hebei (BTH) and Yangtze River Delta (YRD) megaregions compared with the national average in 2000 and 2015.

### Impacts of urban spatial change on air quality

Urban expansion and associated concentration of human activities such as manufacturing, heating and petrol fueled transportation in cities can result in increases in the concentration of pollution. Here, we focus on one particular facet—ambient fine particulate matter with a diameter smaller than 2.5 μm (PM_2.5_), the most predominant air pollutant in many Chinese cities. From 2000 to 2015, urban areas first experienced a dramatic increase of PM_2.5_ concentrations, but then a decrease. Changes in the old and new urban areas followed a very similar pattern to that of the urban area as a whole (Fig. 2C_1_–C_3_). In 2015, the average PM_2.5_ concentration in urban areas was 48.4 μg/m^3^, much higher than the mean of 34.1 for non-urban areas, and the national average of 34.3 μg/m^3^. PM_2.5_ concentrations in 81.1% of the urban areas were greater than 35 μg/m^3^ (the interim target 1 in the World Health Organization's annual air quality guidelines). In contrast, the PM_2.5_ exceedance was 40.8% nationwide. Areas with relatively high PM_2.5_ concentrations were mainly located in densely populated and highly developed East and Central China, and the desert areas in Xinjiang autonomous region (Fig. 5). During 2000–2015 nationwide, a total of 43.6% of the land had a significant increasing trend of PM_2.5_ concentration, mostly located in East and Northeast China where rapid and massive urban expansion occurred (Supplementary Fig. 6). However, 71.3% of the cities had significantly increased PM_2.5_ concentration, but only 2.7% showed a significant decrease.

Changes in urban population and PM_2.5_ concentrations resulted in changes in population exposure to PM_2.5_. Results showed that both the total population exposed to PM_2.5_ > 35 μg/m^3^ and population-weighted exposure to PM_2.5_ concentration had an ‘inverse-U’ trend. The total population exposed to PM_2.5_ > 35 μg/m^3^ was 843 million in 2000, increasing to 1004 million in 2010, but then decreased to 895 million in 2015 (Supplementary Fig. 7). Similarly, the population-weighted exposure to PM_2.5_ concentration was 49.5 μg/m^3^ in 2000, increasing to 52.2 μg/m^3^ in 2005, but then decreased to 35.3 μg/m^3^ in 2015 (Supplementary Fig. 7). However, the total population exposed to PM_2.5_ > 70 μg/m^3^ greatly increased from 11 million in 2000 to 233 million in 2015 (Supplementary Fig. 7).

### Comparisons between old and new urban areas

**Figure 6.**
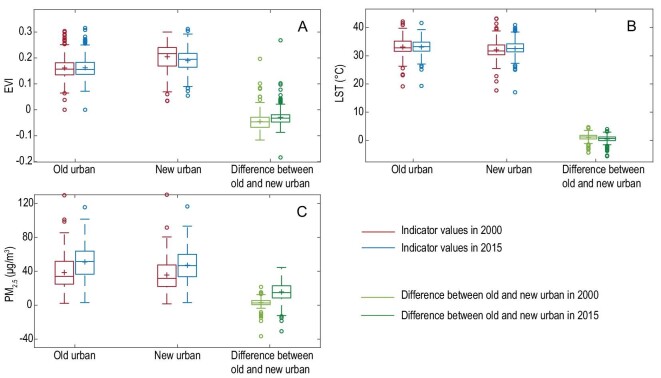
Comparisons of EVI, LST and PM_2.5_ concentration between the old and new urban areas in 2000 and 2015 at the prefectural level. (A), (B) and (C) represent the value distribution of the EVI, LST and PM_2.5_ for all the prefectural cities, respectively. For EVI, the averaged value for the old urban areas in 2015 was 0.162 (standard deviation: 0.043), significantly greater than that of 0.161 (standard deviation: 0.045) in 2000; that for the new in 2015 was 0.190 (standard deviation: 0.046), significantly lower than that of 0.203 in 2000 (standard deviation: 0.053). For LST, the averaged values for the old urban areas in 2000 and 2015 were 33.10 and 33.10, with standard deviations of 2.95 and 2.57, respectively; those for the new in the two years were 32.11 and 32.61, with standard deviations of 3.88 and 3.61, respectively. For PM_2.5_, the averaged values for the old urban areas in 2000 and 2015 were 38.67 and 51.14, with standard deviations of 19.18 and 19.80, respectively; those for the new in the two years were 35.64 and 47.27, with standard deviations of 18.19 and 19.21, respectively. As for LST and PM_2.5_, the differences between 2000 and 2015 for both the old and new were statistically significant (*p* < 0.05).

Environmental impacts differed in newly developed versus older areas of cities. Adverse impacts were prominent in newly urbanized areas, while old urban areas generally showed improved environmental quality (Fig. 2). The environmental quality in newly urbanized areas was still greater overall than that in the old urban areas in terms of EVI, LST and PM_2.5_ concentrations, although the difference between the old and new in general became smaller (Fig. 6). For example, the averaged EVI in the old urban areas were 0.161 and 0.162 in 2000 and 2015, respectively, significantly lower than that of 0.203 and 0.190 in the new. But the difference between the old and new decreased from 0.042 to 0.028.

The proportion of land having increased EVI in old urban areas was 51.5%, which was much larger than the 33.7% in new urban areas (Supplementary Table 3). Consequently, EVI in old urban areas in 2015 overall was significantly greater than that in 2000, suggesting a greening trend in old urban areas. Although a higher proportion of land had significantly increased EVI in old urban areas, the mean values of EVI in the new areas were significantly greater than that in the old. For example, the mean EVI in the new urban areas for all the cities was 0.190 in 2015, significantly higher than that in the old. However, the difference in EVI between the new and the old urban areas became smaller from 2000 to 2015 (Supplementary Fig. 8). This is because in addition to the increase of EVI in old urban areas, the averaged EVI in new urban areas decreased from 0.203 in 2000 to 0.190 in 2015.

The old and new areas of cities had different trends in LST. A higher proportion of land in new urban areas had significant increases in LST (23.4%) than in the old urban areas (8.7%). However, the old urban areas in the majority of cities (73.6%) were warmer than the new ones. In other words, more locations in old urban areas of most Chinese cities had trends of cooling down, but were still warmer than their counterparts of new urban areas (Supplementary Fig. 9). For example, the averaged LST in the old urban areas were 33.1°C in both 2000 and 2015, significantly higher than that of 32.1°C and 32.6°C in the new. But the difference between the old and new decreased from 1.1°C to 0.5°C.

Similar to EVI and LST, PM_2.5_ concentrations in old urban areas were significantly higher than that in new urban areas (Supplementary Fig. 9). In 2015, 82.5% of Chinese cities had higher PM_2.5_ concentrations in old urban areas. In contrast to EVI and LST, more than half of the land (58.1% and 57.6%, respectively) of both the old and new had significant increases of PM_2.5_ concentration. Among the 305 cities, 175 cities (or 57.4%) had more than 50% of land in new urban areas with significant trends of increasing PM_2.5_ concentration. Similarly, 174 cities (or 57.0%) had more than 50% of land in old urban areas with significantly increased PM_2.5_ concentration. Also in contrast to EVI and LST, the difference in PM_2.5_ concentration between the old and new significantly increased from 2000 to 2015 (Fig. 6; Supplementary Fig. 8). This is because while the PM_2.5_ concentrations in both the old and new urban areas increased during this time period, the magnitude of increase in the old urban areas was greater than that in the new, resulting in increased difference.

### Impacts of emergence of urban megaregions

With the emergence of urban megaregions, environmental problems at the city scale expanded to the region level, and gradually became regional issues. For example, with individual cities in a region expanding and forming an urban megaregion, the initially isolated urban heat islands gradually grew into urban heat ‘archipelagos’ (Fig. 4). Taking the Yangtze River Delta urban megaregion as an example, the total area of urban heat islands in the megaregion increased from 10 128 km^2^ in 2000 to 15 270 km^2^ in 2015, and the isolated heat islands expanded and connected to one another (Fig. 4). Similarly, PM_2.5_ concentration in urban megaregions was much higher than the national average, and exhibited clear spatial patterns of clustering (Fig. 5). For example, PM_2.5_ concentrations in 2000 were 34.1 μg/m^3^ and 32.5 μg/m^3^ in the Beijing-Tianjin-Hebei and Yangtze River Delta urban megaregions, respectively, without clear spatial patterns of regional clustering. In 2015, PM_2.5_ concentrations in these two megaregions increased to 52.6 μg/m^3^ and 50.6 μg/m^3^, respectively, showing clear patterns of regional clustering (Fig. 5).

## DISCUSSION

China has experienced rapid, massive, but uneven urbanization since 1978 when the Reform and Opening-up policy began (Fig. 1 and Supplementary Table 1). The urban population in China increased from 172 million, or 17.9% of the population in 1978, to 848 million (60.6%) in 2019. Within the next 10 years, 70.0% of the population, a billion Chinese, will be living in urban areas [26]. Along with this large urban migration in China was the rapid, massive and regionally uneven expansion of urban land within the administrative boundaries of Chinese cities. These transformations are creating daunting environmental challenges for many cities. In fact, the first National Ecosystem Assessment in China shows that nationwide ecosystem services improved from 2000 to 2010, but deteriorating air and water quality, and intensified UHI effects remain grand challenges particularly in cities [27].

### Novel insights and policy implications

Our analysis shows that massive conversion of land to urban uses drives the loss of arable and natural lands (Supplementary Fig. 3, Supplementary Table 2), affects local climate and exacerbates air pollution (Figs 4 and 5, Supplementary Figs 5 and 6). Farmland was the most affected by urban expansion from local to national scales. Although the central government has introduced strict regulations to protect arable land, approximately half of the urban growth at the national scale, and more than 80% in some of the urban megaregions was at the expense of farmland, raising serious concerns about food security [28,29]. The results suggest that national strategies are necessary, but their success relies heavily on local implementation and practices [28].

From 2000 to 2015, urban areas in China as a whole were becoming less green and warmer, and had exacerbated PM_2.5_ pollution (Fig. 2A_1_, B_1_ and C_1_). The impacts of urbanization are particularly prominent in newly urbanized areas, indicating the adverse ecological and environmental impacts of urban expansion (Fig. 2A_3_, B_3_ and C_3_), as documented in many previous studies conducted for individual cities. However, our cross-city comparison analysis, especially the comparison between the old and new urban areas, provides novel insights. First, while overall, urban areas had deteriorating environmental quality, changes in EVI, LST and PM_2.5_ concentrations varied greatly among cities, both in time and in space, suggesting that a ‘one-size fits all’ environmental policy will not work. Rather, city-based policy decisions for environmental quality improvement must be designed. Second, the old urban areas have a general trend in improved ecological and environmental quality, particularly in recent years (Fig. 2A_2_, B_2_ and C_2_). This result reflects the achievement of policies such as ‘Plant Where Possible’ in the old urban areas that have made cities greener and cooler. As many cities in China, especially megacities such as Shenzhen and Shanghai, have been gradually shifting from a growth model of urban expansion to one of internal optimization (i.e. urban renewal), there are enormous opportunities to incorporate ecology into urban design and planning, and policymaking, and introduce green technologies to achieve improved urban sustainability in existing urban areas. Although newly urbanized areas have a tendency to lose vegetation and become warmer because of urban expansion, overall they are greener and cooler than the old urban areas (Supplementary Fig. 9). These results reflect the great efforts of cities dedicated to ecological restoration and environmentally sensitive planning and construction in recent years. Third, the increase of urban size does not necessarily lead to deterioration of environmental quality. Using PM_2.5_ concentration as an example, cities with deterioration of air quality in recent years are typically medium and small-sized (Supplementary Fig. 10). As ‘strictly controlling the size of large cities’ has been frequently used to fight against environment pollution and ecological degradation in China [30], this result suggests the need to move beyond using the size of city as an indicator of appropriate environmental policy. Instead policy must be based on a fuller understanding of the drivers and processes of how urbanization affects environmental quality, and ecosystem structure and functioning across a range of city sizes and aggregations.

### Towards comprehensive understanding of environmental impacts of urban changes

Moving beyond urban expansion, the potential ecological risks of forming urban megaregions warrant further research. According to the National New-Type Urbanization Plan, China's first official plan on urbanization released in 2014, the urban megaregion or urban agglomeration would be the main type of urban spatial form in the next decades [18]. China has proposed building a hierarchical urban megaregion system characterized by five national-level large, nine regional-level medium-sized and six sub-regional-level small-sized urban megaregions [17], and has planned to invest heavily to facilitate the formation and growth of urban megaregions [17]. No doubt more unplanned urban megaregions will be emerging and expanding in the coming decades. Although the social and economic challenges and opportunities of urban megaregions have been widely discussed [17,18,31], little is known about the potential ecological risks of such massive, spatially connected land of development. Our results show that in some of the existing and emerging urban megaregions, air pollutants were highly clustered (Fig. 5), and isolated urban heat islands of individual cities increasingly became connected, forming urban heat archipelagos (Fig. 4). These results indicate that forming urban megaregions might result in severe environmental problems with magnitudes and extents well beyond the individual city scale, and therefore become much more difficult to solve. With continuous urban growth and densification in certain regions, in addition to climate change, the potential ecological risks of urban megaregions must be carefully evaluated, and advanced planning for mitigation and adaptation strategies are required.

In addition to urban expansion, our analysis shows that cities can have very high internal dynamics (Supplementary Fig. 2). Such changes may have significant ecological and environmental impacts. For example, our results show that the old urban areas in general were turning greener (Figs 2A_2_ and 3, and Supplementary Fig. 9A_1_), and becoming less warm in a large proportion of locations, especially in recent years (Supplementary Fig. 8B). These changes are likely related to the great efforts that Chinese cities have dedicated to urban greening [32,33]. Although the causes of internal city dynamics are very complex, such changes are largely a result of redevelopment of the large number of urban villages built during early phases of rapid urbanization [34], the relocations of industries and redevelopment [35], and the infill of ‘left over’ land previously unbuilt [35,36]. Internal city dynamics are observed in both growing and shrinking cities worldwide [23,37]. Although its social and economic impacts have been widely examined [38,39], much less is known about the ecological and environmental consequences, in contrast to the numerous studies that have examined the ecological and environmental impacts of urban expansion. While internal city dynamics may possess ecological and environmental challenges such as loss of native species, generating noise and temporally increasing air pollution [40], such changes also provide enormous opportunities to introduce sustainable technologies and practices to make the city greener, more livable, resilient, and energy and resource efficient [28,41]. To fully understand how urbanization affects ecosystem structure and functioning, we need to move beyond urban expansion to understand the ecological consequences of internal city dynamics. Such understanding can provide important insights on urban planning and design, and policy-making that aim to minimize the adverse environmental impacts caused by internal city dynamics, and maximize the social and ecological benefits. The limit of data availability meant that we focused only on the within-city changes of greenspace in nine big cities in China as examples. It would be interesting to explore further whether the findings from the nine big cities can be applied to small and medium-sized cities in China, or even broadly to cities worldwide.

### Turning urbanization into an opportunity for sustainable development

Although urbanization has long been seen as one of the major drivers of ecological degradation, and thereby as a major obstacle to sustainable development [6,41], it can, and must be turned into an opportunity for sustainable development [28,40–43]. Our results that EVI far from urban centers had a significantly increasing trend exemplify the potential of urbanization for better protecting our Earth (Figs 2A_4_ and 3, and Supplementary Table 3). The increase of EVI far from urban centers is a regional outcome of the establishment of new urban cores and the growth and densification of existing cores. Urban expansion, growth and densification are direct results of China's national urbanization policy, and can drive increase in non-urban EVI in three ways. First, the policy has resulted in massive emigration of people from rural villages and small towns to cities. Many of these people had been employed in agriculture, forest products and grazing. Their departure from the countryside has likely reduced the amount and intensity of land management outside of cities. Consequently, vegetation cover has increased in non-urban lands. Second, people who remain in rural areas are less dependent on earning a livelihood from agricultural and related employment. People who continue to live in rural areas may depend more on pensions or on remittances from family members who have emigrated to cities or larger towns. Third, lifestyle shifts in the countryside may accompany the regional redistribution of population to urban centers. The consumption habits of family members and friends who have moved to cities may influence those who remain in rural areas. In addition, remaining rural residents may be in a better position to follow such urban influences given that release from agricultural employment increases the time they have for leisure activities. In general, lifestyles in rural areas may begin to follow urban models, relying on media, investment in luxury goods and upgraded housing. The increase in EVI outside of the growing cities is therefore a reflection of the growing connectivity expected and observed in urban megaregions worldwide. Currently these specific expectations are hypotheses that must be tested in further social-ecological research.

New regional research can be guided by a conceptual model, the continuum of urbanity [44], which emphasizes that urban and rural social and ecological characteristics can in fact mix in many places in a region. Regional connectivity is the foundation of the continuum [6]. The continuum of urbanity identifies four dimensions of urban-rural interaction. One is livelihood, or how people support themselves and operate in formal, informal, local and global economies. The second dimension is lifestyle, or how people identify themselves in terms of social group and how they represent this identity through their consumption and leisure activities. The third dimension is the nature of connectivity, that is, the material pathways and electronically mediated transfers that people use. The dimension of connectivity includes such things as commuting, seasonal or permanent migration, social media, or the transfer of information, goods and financial capital. Finally, the first three processes strongly influence the specific places where people live, work and travel. That is, the continuum is expressed differently in specific places. Of course, the ecological and social structures and functions in specific places will also influence how people engage with the other three dimensions. Resources, environmental and social constraints, and hazards are all spatially anchored and together characterize the particular urban nodes, habitats or social-ecological systems that are arrayed along the conceptual continuum. The EVI trends discovered here are an example of functions that must be understood regionally in the context of the continuum of urbanity. The continuum suggests the things that must be measured to understand the functioning of urban megaregions and help evaluate their effects on global sustainability.

## MATERIALS AND METHODS

### Data

We used China land cover classification maps obtained from the China National Ecosystem Assessment program [27]. The maps were derived from the 30 m resolution Landsat TM data. We used maps for the mainland of China in 2000 and 2015, and maps for the six urban megaregions from 1980 to 2015, with four time slices. The land cover classification data have six categories—forest, grass, farmland, water, developed land and bare ground, where developed land consists of residential, commercial, industrial and transportation lands in both urban and rural areas. We also used two types of high spatial resolution imagery data from SPOT-5 (Systeme Probatoire d’Observation de la Terre) and ALOS (Advanced Land Observation Satellite) to quantify the internal city dynamics for nine major cities in China—Beijing, Tianjin, Tangshan, Shanghai, Nanjing, Hangzhou, Changzhou, Suzhou and Wuxi. Four land cover types—impervious surface, vegetation, water and bare soil—were identified and classified using a combination of SPOT-5 and ALOS imagery data for the years of 2005 and 2010 [37].

We used the Enhance Vegetation Index (EVI) vegetation layer from MOD13A2 Version 6 product, which is derived from MODIS/Terra. Compared to the Normalized Difference Vegetation Index (NDVI), EVI has improved sensitivity over high biomass regions. The dataset comprises 16-day composites and the spatial resolution is 1 km (available at: https://lpdaac.usgs.gov/products/mod13a2v006/). Based on the quality assurance (QA) layer for each 16-day composite EVI layer, all the reliable pixels were selected and then were used to calculate the mean value of EVI in each year from 2000 to 2015. By stacking all layers with a mean value of EVI, we constructed the annual EVI time-series dataset for the latter trend analysis.

We used LST datasets from MOD11A2 Version 6 product, which provides an average 8-day per-pixel LST with a spatial resolution of 1 km (available at: https://lpdaac.usgs.gov/products/mod11a2v006/). We used the daytime products and calculated LST for the summer of each year (from 1 June to 31 August) from 2000 to 2015.

We used both remotely sensed annual mean and ground operational measured PM_2.5_ concentrations. The annual mean concentration of PM_2.5_ from 2000 to 2015 was obtained from the Atmospheric Composition Analysis Group at Dalhousie University (available at: http://fizz.phys.dal.ca/∼atmos/martin/). The product was derived with an optimal estimation algorithm that combines MODIS observation and Geos-Chem chemical transport model estimation [45,46]. Their latest data version (V4.CH.02) for China, which was further combined with geophysical-statistical estimates using the recently expanded PM_2.5_ measurements network in China from May 2014 to December 2016, was used in this research [45,46]. The product has an approximately 1 km resolution in China. We also used the ground operational measured concentration of PM_2.5_ during 2015–2018 from China's Urban Air Quality Monitoring Network (CUAQMN) for 333 Chinese cities. The ground monitoring network collects data on PM_2.5_ concentration every hour, and we used the annual average in our analysis.

### Multi-scale quantification of urban expansion

Although numerous studies have documented urban expansion of individual cities, or at a regional scale, few have quantified and detailed the magnitude and spatial patterns of urban expansion for the whole country at the multi-scales of city, region and nation using 30 m spatial resolution data. We quantified the spatial and temporal patterns of urban expansion of China at multiple scales based on the developed land extracted from the land cover maps from 2000 to 2015. Two commonly used indices, newly developed area (*A*_new_) and growth rate (GR) of developed land, were used for assessing the magnitude and speed of urban expansion, respectively [47]. The calculations were showed as follows:

(1) $$\begin
{equation}
{A_{\rm new}} = {A_{\rm end}} - {A_{\rm start}},
\end{equation}$$

(2) $$\begin
{equation}
\textit{GR} = \frac{{{A_{\rm new}}}}{{{A_{\rm start}}}} \times 100\% ,
\end{equation}$$

where *A*_start_ and *A*_end_ represent the area of developed land in 2000 and 2015, respectively. We performed the calculations at the national, regional and prefectural city scales. In our study, the regional scale included four geographical regions: northeast, east, central and west (Supplementary Table 1). Additionally, we also quantified the spatial and temporal patterns of urban expansion in the six urban megaregions from 1980 to 2015.

### Old and new urban comparison

The urban boundary for each prefectural city in 2000 and 2015 was delineated based on the classification data of developed land, followed the method detained in Hu *et al.* [9]. Briefly, we first generated grids with a size of 900 m × 900 m based on the 30 m land cover classification data. We used a size of 900 m that is 30 times of the spatial resolution of the 30 m land cover classification data, and also approximately equal to the spatial resolution of the 1 km of the data sources for EVI, LST and PM_2.5_. We then calculated the proportional cover of developed land in each grid. We identified all the grids with more than 50% developed land, and dissolved them, resulting in one large polygon and many scattered smaller ones. We then removed the scattered ones that did not connect to the large polygon and dissolved the grids with less than 50% of developed land but totally encompassed by the large polygon. We defined the areas within the urban boundary delineated in 2000 as the old urban area, and the urban area developed between 2000 and 2015 as the new urban area. Areas outside of the urban boundary were defined as non-urban (Supplementary Fig. 11). Using these urban boundaries, we compared the spatial patterns of EVI, LST and PM_2.5_ concentration, and their changes, including the annual mean value and its differences, from 2000 to 2015 among the urban areas as a whole, non-urban areas, old urban areas and new urban areas. We further compared the proportional cover of land with different trends of EVI and LST derived from trends analysis. In addition, we standardized the values of the three indicators by population using a population-weighted approach for year 2000 and 2015 [48] (Supplementary Fig. 12). Results from the standardized indicators were slightly different from the non-standardized ones, but had similar patterns (Supplementary Fig. 13).

### Trends analysis

Trends of EVI and LST from 2000 to 2015 were calculated by Ordinary Least Square (OLS) regressions based on time series data of annual mean. Taking EVI as an example, we estimated the trend of change for each pixel by calculating the slope of the OLS regression model:

(3) $$\begin
{equation}y = ax + b,\end{equation}$$

where *x* is the annual mean value of EVI derived from the 16-day MODIS composite layers, *a* is the slope of the linear model, representing the trends of EVI, and *b* is the intercept. We consider that the trend is statistically significant when *a* is significantly different from 0 (*p* < 0.05), where *a* greater than 0 means a trend of increase, and less than 0 means a trend of decrease. Based on the value of slope in each pixel with spatial resolution of 1 km, we mapped out the spatial patterns of the EVI trends and quantified the proportional cover of land with different trends for the entire national, urban and non-urban areas, and the old and new urban areas.

### Ecological and environmental impact analysis

We calculated the land cover transfer matrices based on the land cover data in different years to quantify the land conversion caused by urban expansion. Using the land cover transfer matrices, we first calculated the area and its proportion to the total of each land cover type that was converted to developed land for the entire nation, different urban megaregions and different cities. We further calculated the ratio of the area of land converted to developed land to the total area of land conversion for each land cover type, and then used the ratio to evaluate the contribution of urban expansion on the conversion of a certain type of land cover.

We used the intensity of urban heat island (UHII) and its change to evaluate the impacts of urban expansion and internal city dynamics on local climate. The intensity of UHI was the LST difference between the urban area and the non-urban. Using the urban boundaries defined above, we calculated the UHII in years 2000 and 2015, separately. We also calculated UHII for the old urban and the new urban.

The population exposure to PM_2.5_ was estimated by overlaying the remotely sensed PM_2.5_ concentration data layer with the population data layer. We used the LandScan^TM^ population distribution product created by the Oak Ridge National Laboratory (ORNL), USA. This population data has an approximately 1 km spatial resolution at global scale, and we used a subset of the product that covers China in 2000, 2005, 2010 and 2015. We calculated the population exposure to annual PM_2.5_ concentration greater than 35 μg/m^3^, which is the Interim Target-1 (IT-1) of the World Health Organization's (WHO’s) Air Quality Guideline (AQG), and 70 μg/m^3^, which is twice IT-1 [49]. Additionally, the population weighted PM_2.5_ concentration in China was also calculated with remotely sensed PM_2.5_ and gridded population density for the years 2000, 2005, 2010 and 2015, followed the method detailed in van Donkelaar *et al.* [46].

## DATA AVAILABILITY

All data needed to evaluate the conclusions in the paper are present in the paper and/or the Supplementary data. Additional data related to this paper may be requested from the authors for non-commercial use.

## Supplementary Material

nwab107_Supplemental_File
