# Supplementary material for: Beyond city expansion: multi-scale environmental impacts of urban megaregion formation in China
Source: Natl Sci Rev. 2021 Jun 22;9(1):nwab107. doi: 10.1093/nsr/nwab107 (PMC8776543; doi:10.1093/nsr/nwab107)
Supplement: nwab107_Supplemental_File [file nwab107_supplemental_file.docx]

Supplementary Materials for

**Beyond city expansion: multi-scale environmental impacts of urban megaregion formation in China**

Weiqi Zhou, Wenjuan Yu, Yuguo Qian, Lijian Han, Steward T. A. Pickett, Jing. Wang, Weifeng. Li, Zhiyun Ouyang*

* Corresponding author, Email: zyouyang@rcees.ac.cn

**This file includes:**

Fig. S1. Urban expansion in six planned urban megaregions.

Fig. S2. The change of greenspace for different size of patches and the magnitude and percentage of greenspace change in the urban core areas.

Fig. S3. Land cover change caused by urban expansion in China and the six urban megaregions.

Fig. S4. Farmland fragmentation in six planned urban megaregions.

Fig. S5. Changes in UHI intensity from 2000 to 2015 in the Yangtze River Delta (YRD) urban megaregion.

Fig. S6. Spatial pattern of changes in PM_2.5_ concentration in China from 2000 to 2015.

Fig. S7. Population exposure and population weighted PM_2.5_ concentration.

Fig. S8. The annual differences in EVI, LST and PM_2.5_ between the old and new urban areas from 2000 to 2015.

Fig. S9. Difference in EVI, LST, and PM_2.5_ and changes in the old and new urban areas for all the prefectural cities.

Fig.S10. Relationship between urban size and PM_2.5_ concentration.

Fig. S11. Concept of China’s urban expansion and the comparison of ecological changes between old and new urban areas.

Fig. S12. Population and total area of the old and new urban areas in 2000 and 2015.

Fig. S13. Population-weighted EVI, LST and PM_2.5_ concentration in 2000 and 2015 at the prefectural level.

Table S1. Urban growth at multiple scales from 2000 to 2015.

Table S2. The contribution of urban expansion to land cover change for the whole nation, and the six urban megaregions.

Table S3. Area and proportional cover of the EVI trends.

Table S4. Area and proportional cover of the LST trends.

**Figures**

**Fig. S1. Urban expansion in six planned urban megaregions.** The panel in the left shows the spatial location of the six urban megaregions, and the right panel shows the proportional cover of developed land in the six urban megaregions and the change from 1980 to 2015, indicating large variations in the magnitude and expanding rate among urban megaregions.


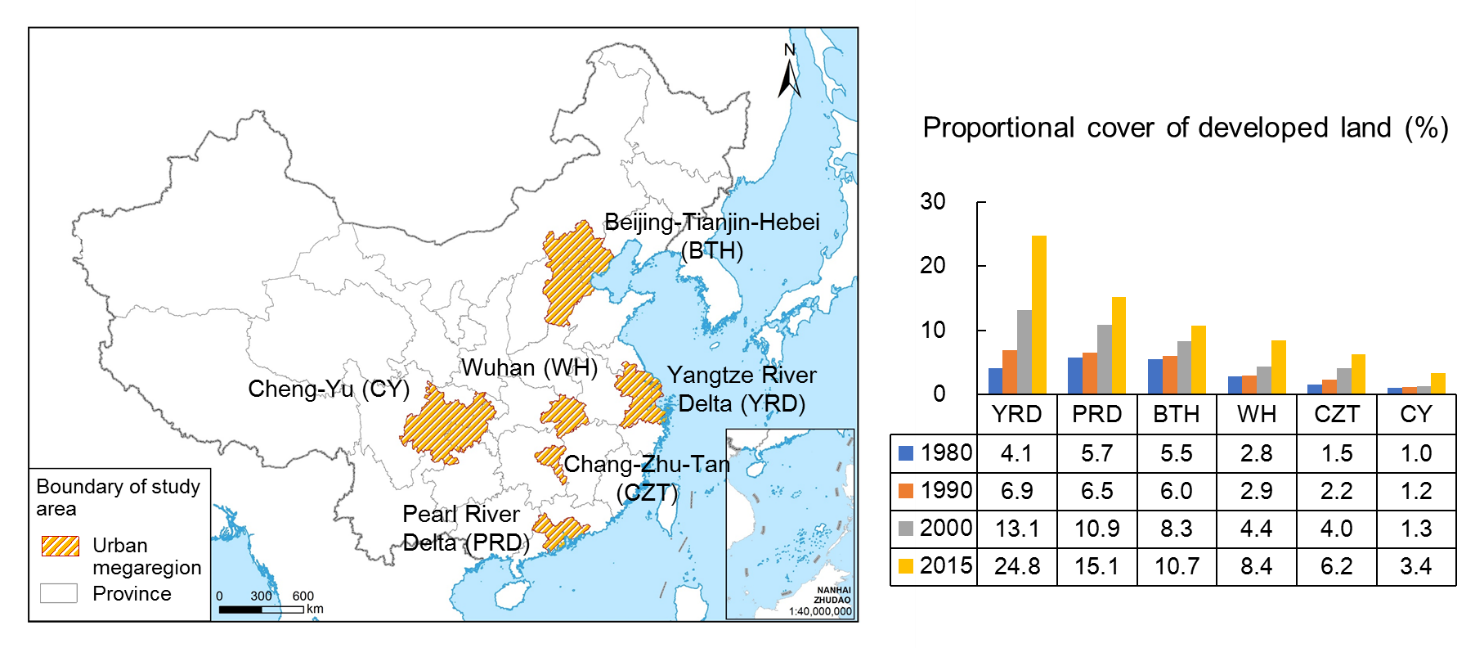


**Fig. S2. The change of greenspace for different size of patches and the magnitude and percentage of greenspace dynamics in the urban core areas.** Panel **A**, changes in greenspace within the 5^th^ ring road areas in Beijing, derived from high-resolution imagery acquired from SPOT (2.5 m) in 2005 and AOLS (2.5 m) in 2009; gray and green colors represent non-greenspace and greenspace with no change, red color represents lost greenspace, and blue color represents gained greenspace. Panel **B**, examples of the greenspace change, small or large. Panel **C**, The magnitude and percentage of greenspace dynamics in the urban core areas of nine major cities from 2005 to 2010.


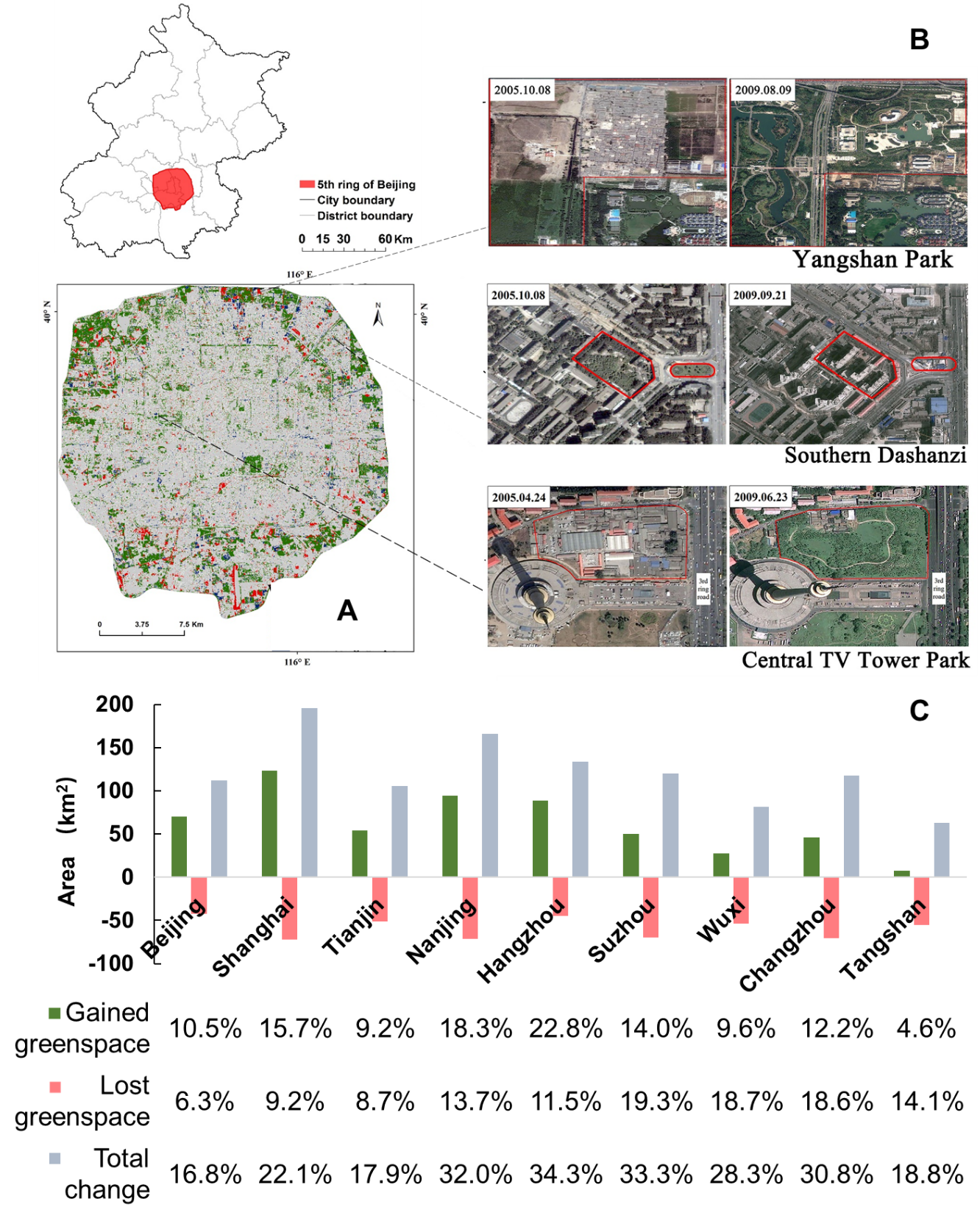


Data adopted from Zhou et al. 2018, *Sci Total Environ.*

**Fig. S3. Land cover change caused by urban expansion in China and the six urban megaregions.** Panel **A** shows the proportions of different types of land cover converted to developed land in the whole country in 2000-2015; Panel **B** shows the proportions of different types of land cover converted to developed land in the six urban megaregions from 1980 to 2015.


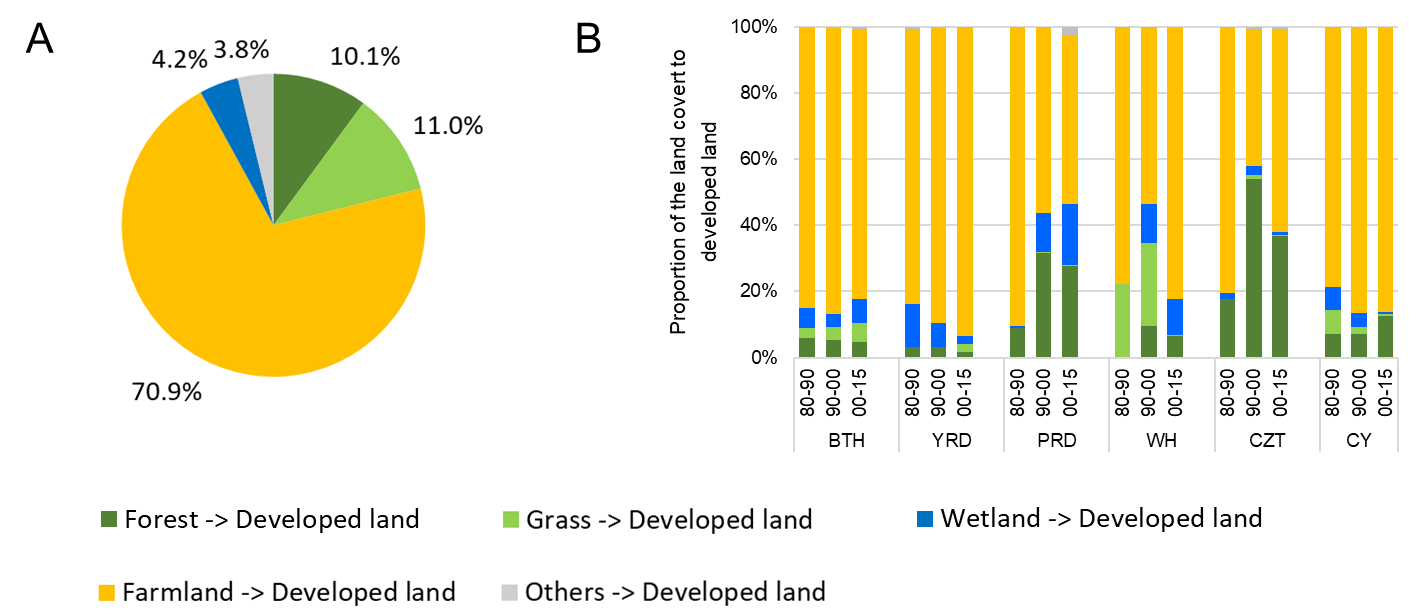


**Fig. S4. Farmland fragmentation in six planned urban megaregions.** The abbreviations of the six urban megaregions are the same as that in Fig.S1. Panels **A** and **B** show the decrease of mean patch size and increase of patch density of farmland in the six urban megaregions, indicating increased fragmentation of farmland from 2000 to 2015.


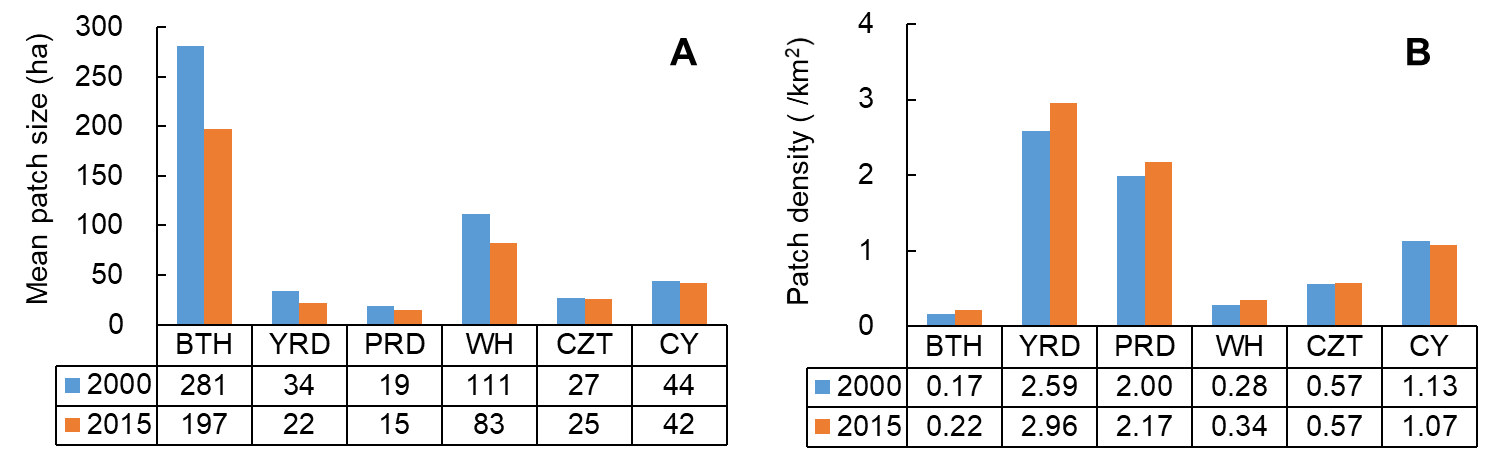


**Fig. S5. Changes in UHI intensity from 2000 to 2015 in the Yangtze River Delta (YRD) urban megaregion.** Panels **A** and **B** show the UHI intensities in 2000 and 2015, respectively. Panel **C** shows changes in UHI intensity from 2000 to 2015. Panel **D** shows the trend of LST from 2000 to 2015, indicating that the decrease of UHI intensity in some of cities in the YRD urban megaregion was caused by the warming of the surrounding regions of the cities, but not the cooling of the cities.


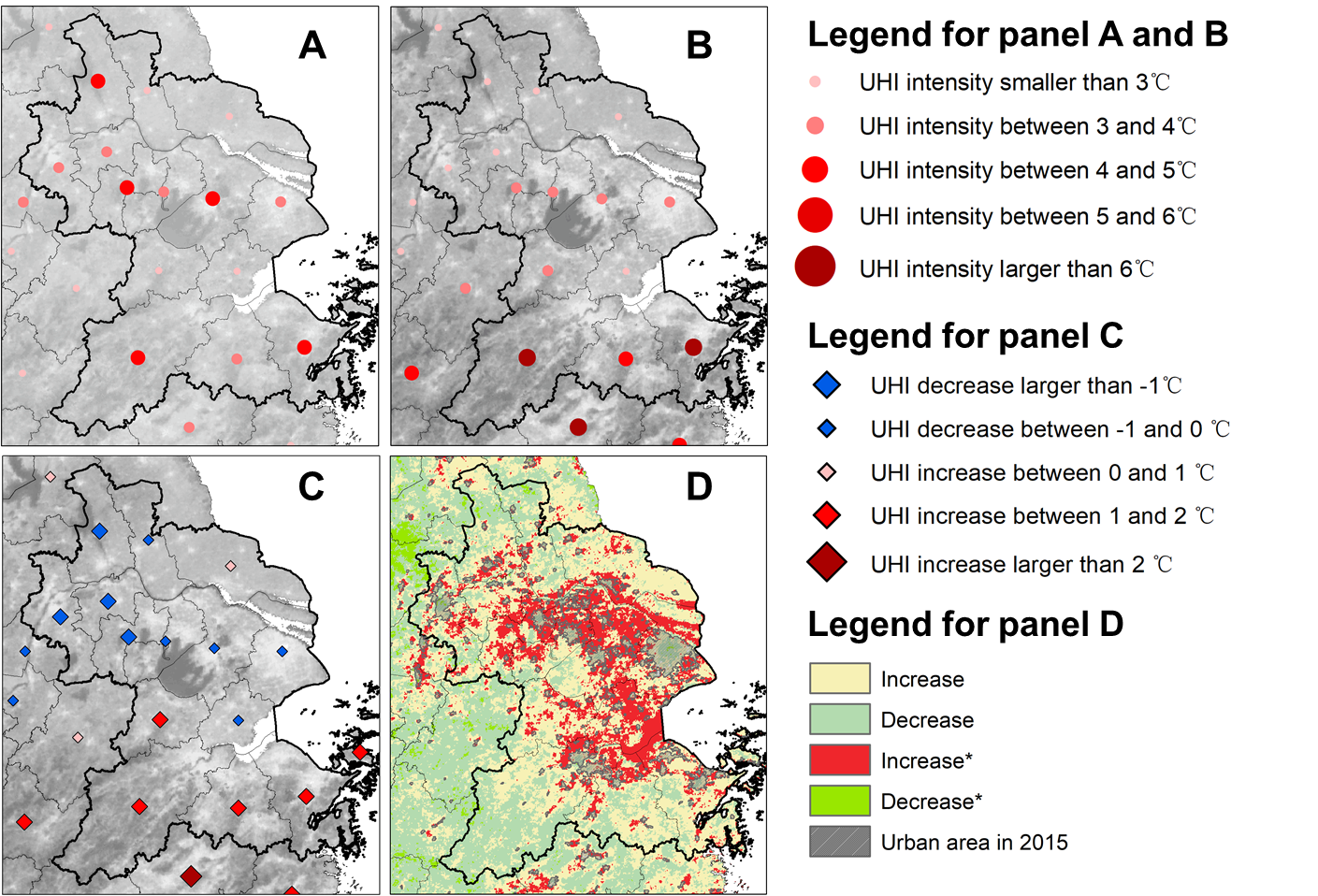


**Fig. S6. Spatial pattern of changes of PM_2.5_ concentration in China from 2000 to 2015.** 87.1% of the land had trends of increase, and 43.6% were significant.

**
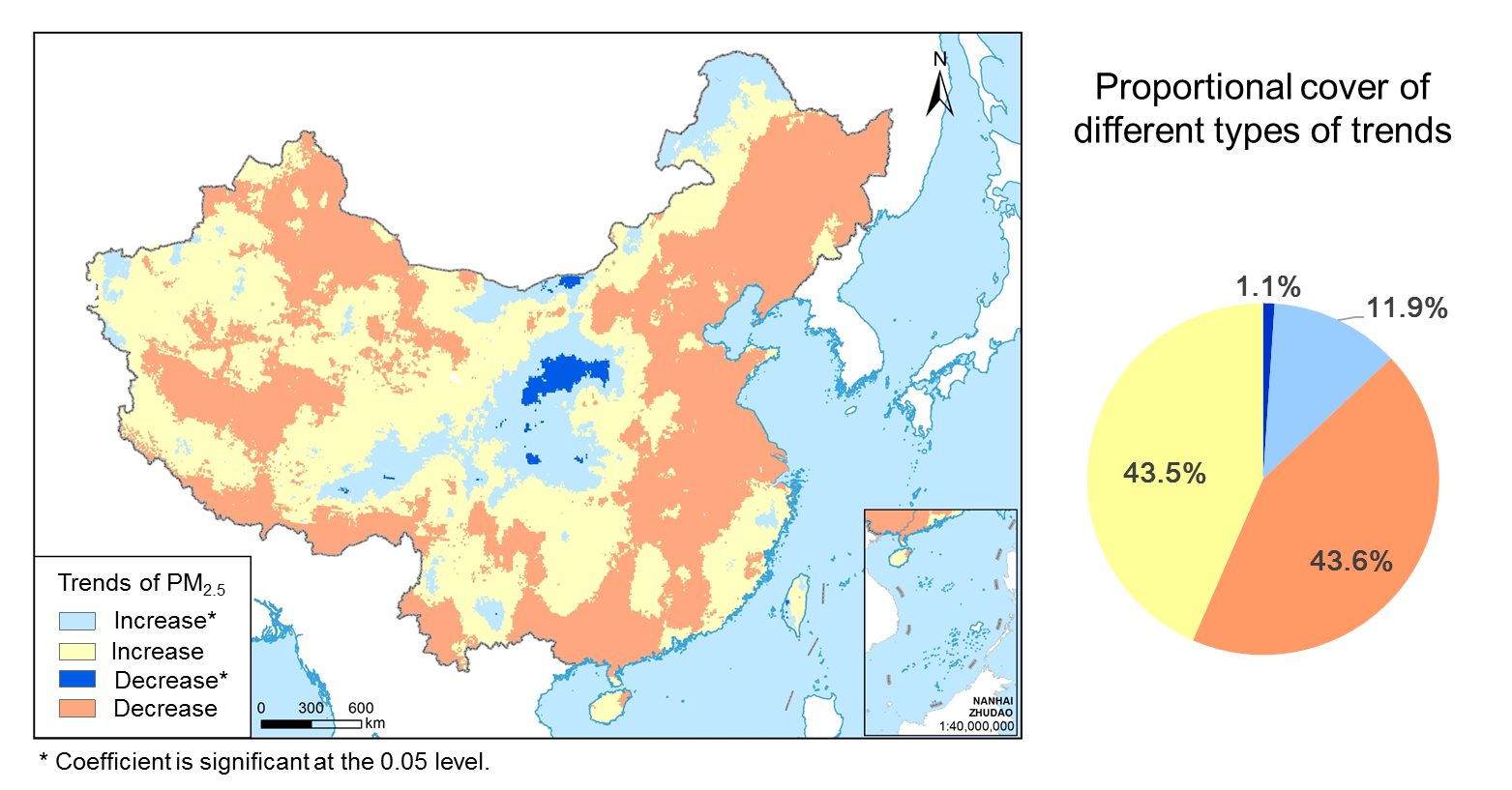
**

**Fig. S7. Population exposure and population weighted PM_2.5_ concentration.**


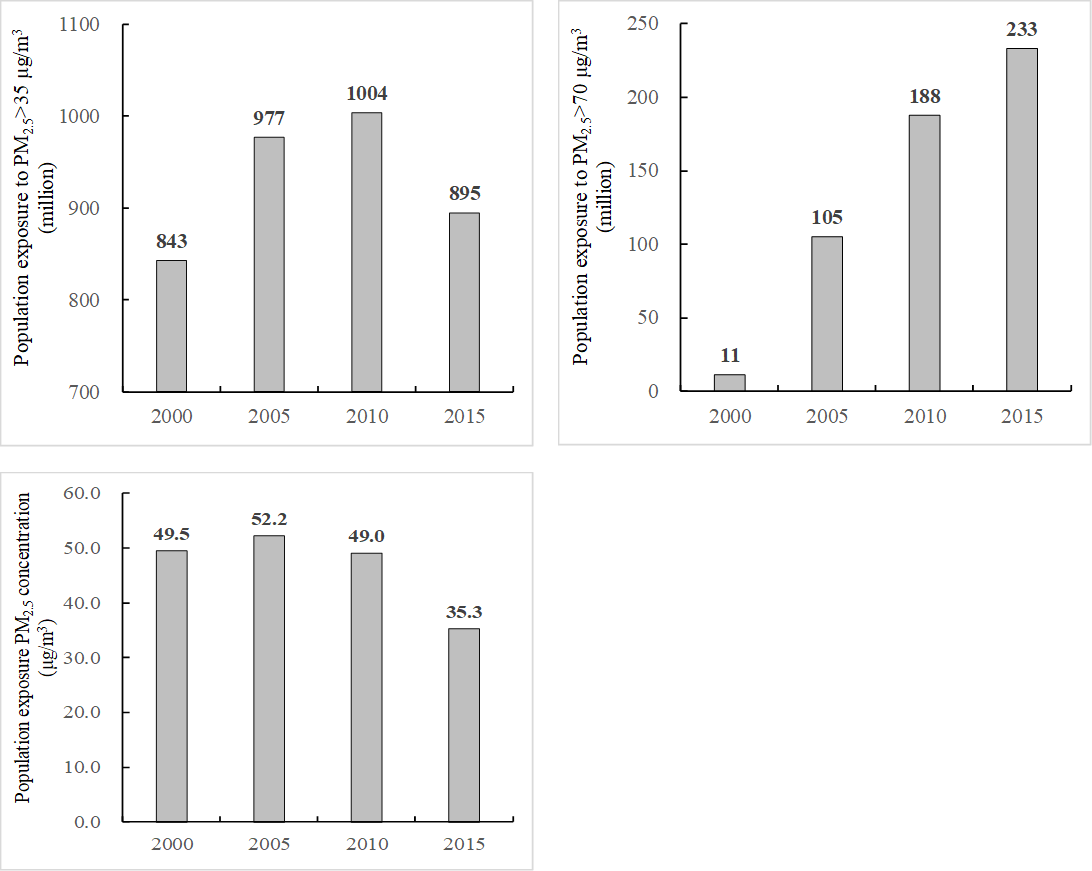


**Fig. S8. The annual differences in EVI, LST and PM_2.5_ between the old and new urban areas from 2000 to 2015.** Panel **A,** a significant trend of decline in the difference of EVI between the old and new urban areas; panel **B**, a significant trend of decline in the difference of LST between the old and new urban areas; panel **C,** a significant trend of increase in the difference of PM_2.5_ concentration between the old and new urban areas.


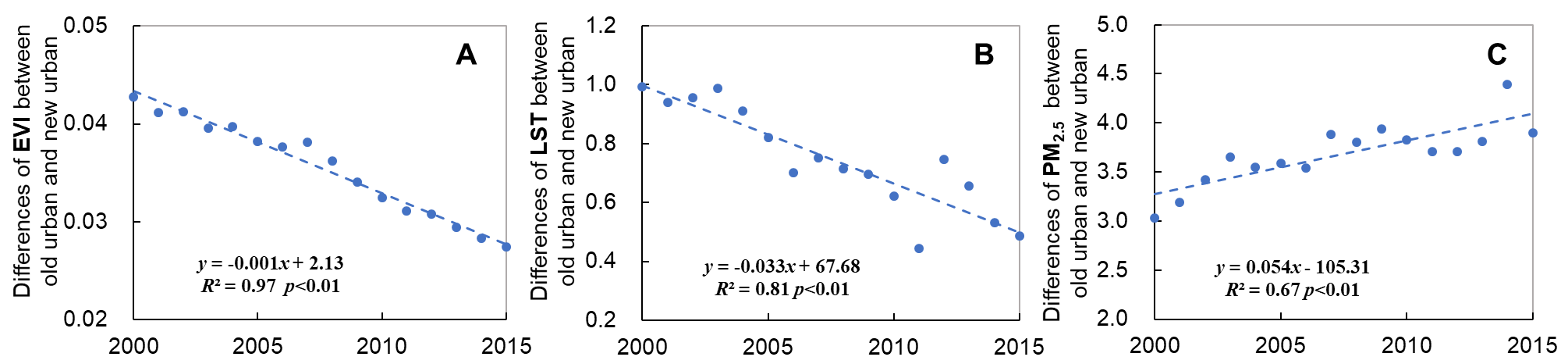


**Fig. S9. Difference in EVI, LST, and PM_2.5_ and changes in the old and new urban areas for all the prefectural cities.** Panels **A_1_** and **A_2_** show that EVI in the newly urbanized areas of most cities was larger than that in the old, but proportional cover of the old urban areas with increased EVI was higher than that in the new; panels **B_1_** and **B_2_** show that LST in the newly urbanized areas of most cities was lower than that in the old, but proportional cover of the new urban areas with increased LST was higher than that in the old; panels **C_1_** and **C_2_** show that PM_2.5_ concentration in the newly urbanized areas of most cities was lower than that in the old, and proportional cover of the new urban areas with increased PM_2.5_ concentration was about the same as that of the old.


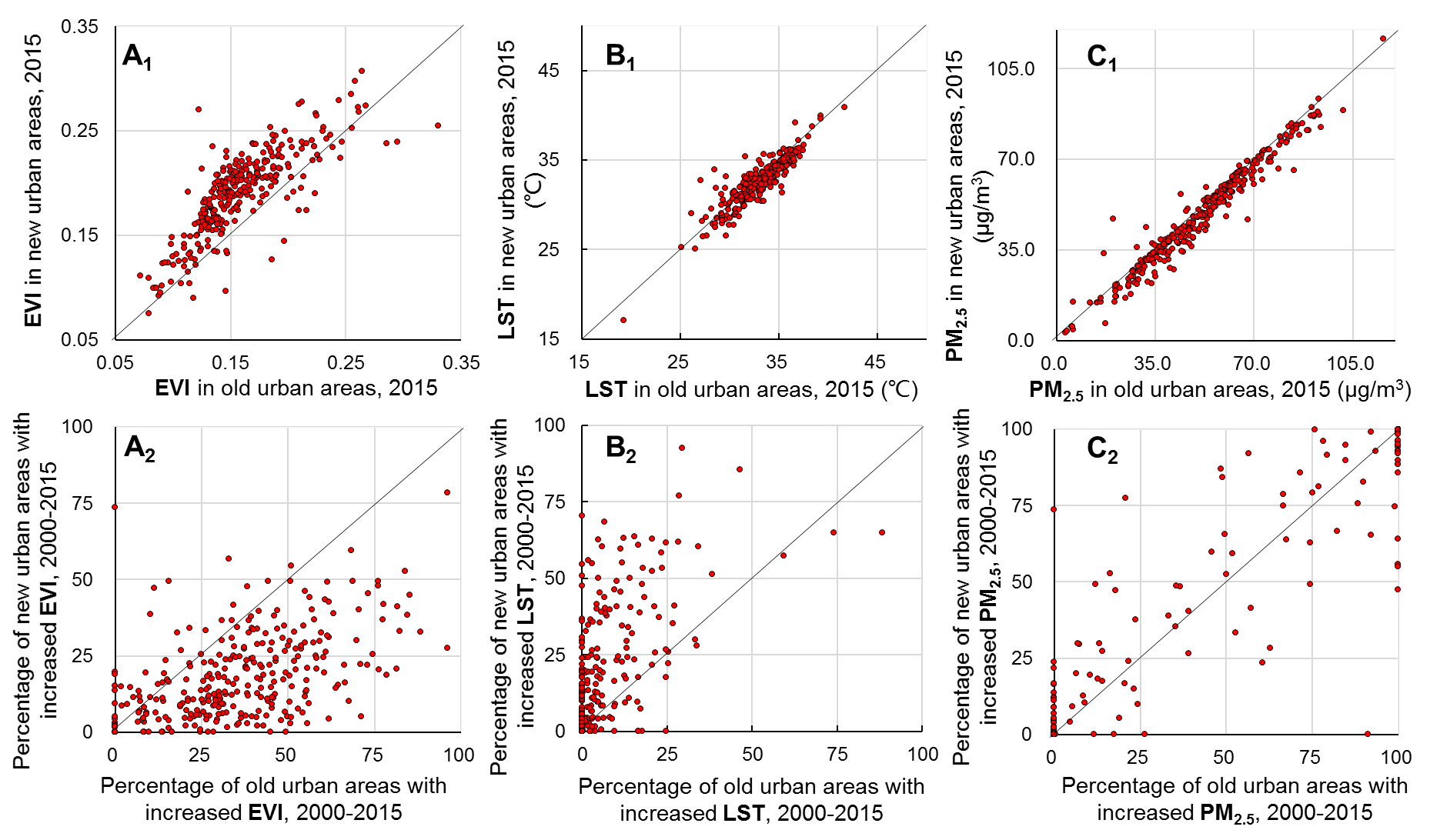


**Fig.S10. Relationship between urban size and PM_2.5_ concentration.** Blue circles represent cities with decreased PM_2.5_ concentration and red ones for cities with decreased PM_2.5_ concentration. The size of the circle measures the magnitude of change in PM_2.5_ concentration. Panel **A** shows the relationship between the size of built-up area and changes in PM_2.5_ concentration, showing increase mostly occurred in small and medium sized cities; panels **B** and **C** also show that increase of PM_2.5_ concentration mostly occurred in small and medium sized cities in terms of population and GDP.


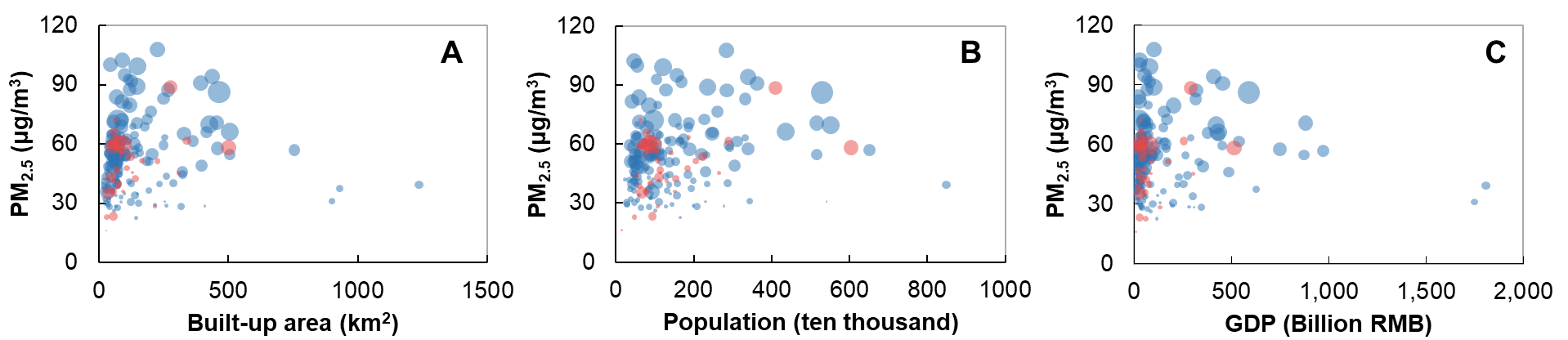


**Fig. S11. Concept of China’s urban expansion and the comparison of ecological changes between old and new urban areas.** Exemplified by Beijing, the dark red represents old urban areas that were developed before 2000, while the blue for new urban areas that were developed from 2000 to 2015. On the comparison between status in old and new urban areas, a city located in the green part of the graph indicates that its new urban area has higher values of EVI, LST and PM_2.5_ concentration, compared with the old urban. On the comparison between changes in old and new urban areas, a city located in the green part of the graph indicates the new urban has greater changes in EVI, LST and PM_2.5_ concentration, compared with the old urban.


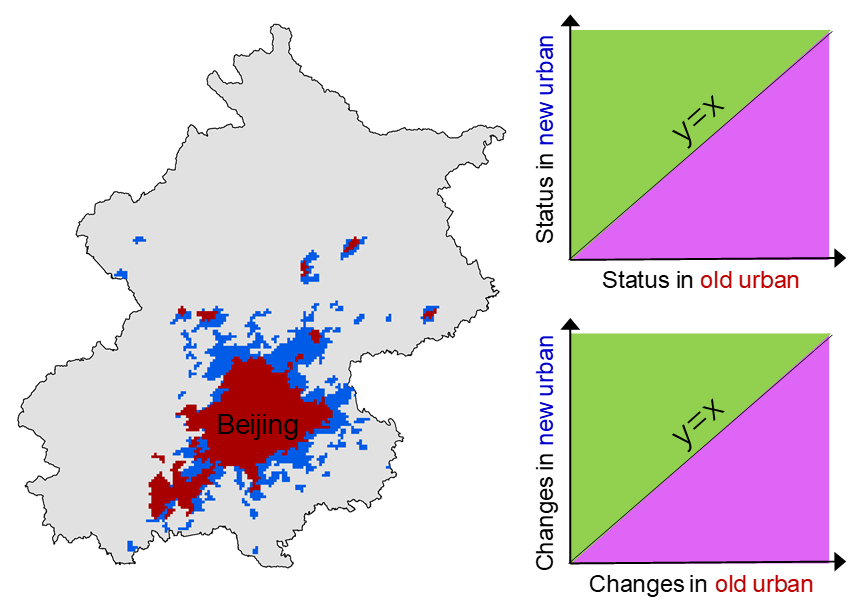


**Fig. S12. Population and total area of the old and new urban areas in 2000 and 2015.** Panel **A**, the population in the old and new urban areas generated based on the LandScan^TM^ data. The total population in the old and new urban areas were 304.5 and 217.2 million in year 2000, and were 406.0, and 268.3 million in year 2015, respectively. Panel **B**, the areas of the old and new urban areas. The total areas of the old and new urban were 2.6×10^4^ km^2^ and 4.5×10^4^ km^2^, respectively.


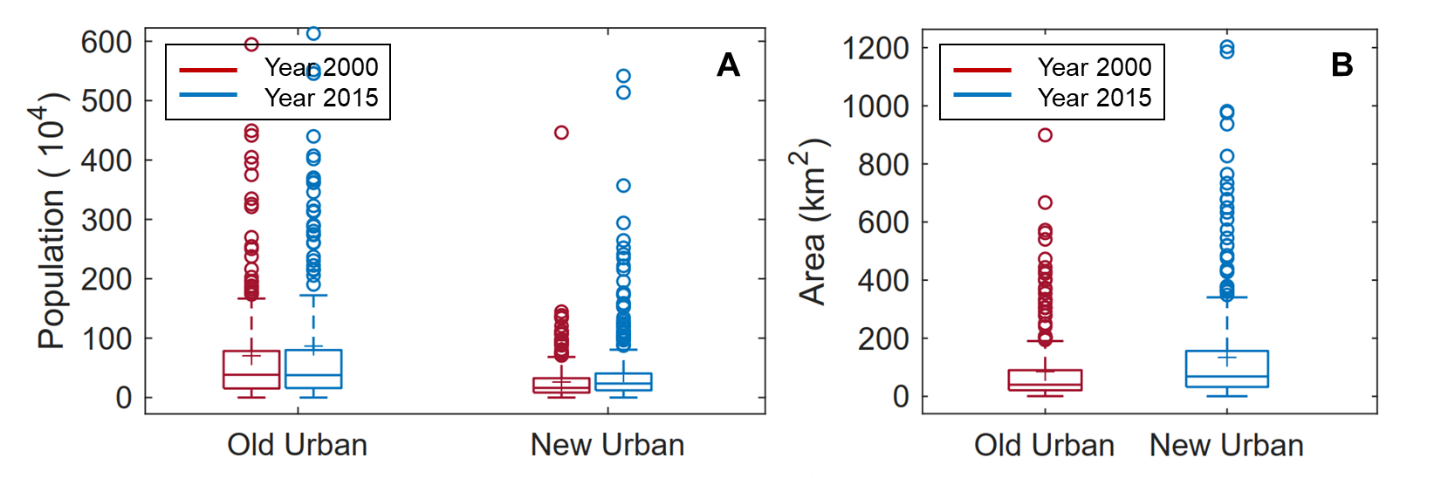


**Fig. S13. Population-weighted EVI, LST and PM_2.5_ concentration in 2000 and 2015 at the prefectural level**. Panels **A**, **B** and **C** are the population-weighted values for the three indicators.


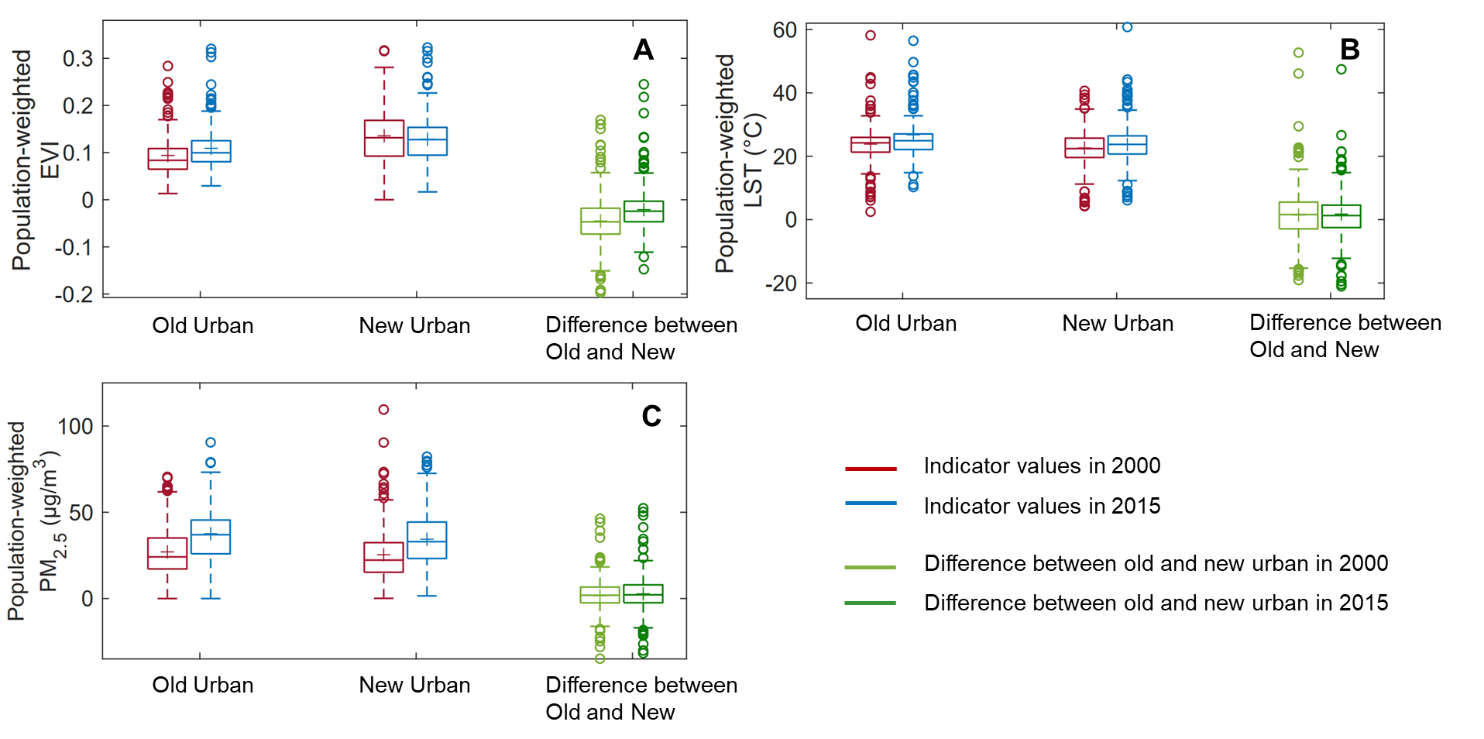


**Tables**

**Table S1.** **Urban growth at multiple scales from 2000 to 2015.**

| Region | Developed land in 2000 (km^2^) | Developed land in 2015 (km^2^) | Newly developed land in 2000-2015 (km^2^) | Growth rate (%) | City | Developed land in 2000 (km^2^) | Developed land in 2015 (km^2^) | Newly developed land (km^2^) | Growth rate (%) |
| --- | --- | --- | --- | --- | --- | --- | --- | --- | --- |
| Country | 211 756  (2.2) | 291 747  (3.0) | 79 991 | 37.8 | – | – | – | – | – |
| North-East | 28 066  (3.5) | 32 551  (4.1) | 4 486 | 16.0 | Shenyang | 1 662.1 | 2 053.6 | 391.4 | 23.5 |
|  |  |  |  |  | Changchun | 1 895.7 | 2 234.5 | 338.8 | 17.9 |
|  |  |  |  |  | Harbin | 1 884.8 | 2 154.9 | 270.2 | 14.3 |
| East | 79 295  (8.6) | 113 680  (12.4) | 34 385 | 43.4 | Haikou | 107.2 | 213.5 | 106.3 | 99.2 |
|  |  |  |  |  | Nanjing | 938.6 | 1 705.4 | 766.7 | 81.7 |
|  |  |  |  |  | Hangzhou | 1 063.5 | 1 712.3 | 648.8 | 61.0 |
|  |  |  |  |  | Tianjin | 1 805.0 | 2 877.4 | 1 072.5 | 59.4 |
|  |  |  |  |  | Shanghai | 1 884.0 | 2 785.1 | 901.1 | 47.8 |
|  |  |  |  |  | Fuzhou | 728.3 | 1 023.4 | 295.1 | 40.5 |
|  |  |  |  |  | Beijing | 2 154.4 | 2 993.8 | 839.4 | 39.0 |
|  |  |  |  |  | Jinan | 1 303.8 | 1 759.8 | 456.0 | 35.0 |
|  |  |  |  |  | Guangzhou | 1 209.9 | 1 585.6 | 375.8 | 31.1 |
|  |  |  |  |  | Shenzhen | 656.2 | 830.3 | 174.2 | 26.5 |
|  |  |  |  |  | Shijiazhuang | 1 621.6 | 1914.9 | 293.3 | 18.1 |
| Center | 54 138  (5.2) | 73 608  (7.2) | 19 471 | 36.0 | Wuhan | 861.8 | 1 720.1 | 858.3 | 99.6 |
|  |  |  |  |  | Hefei | 1 112.4 | 2 174.2 | 1 061.7 | 95.4 |
|  |  |  |  |  | Zhengzhou | 1 029.2 | 1 820.6 | 791.4 | 76.9 |
|  |  |  |  |  | Changsha | 598.3 | 909.3 | 311.0 | 52.0 |
|  |  |  |  |  | Nanchang | 653.4 | 896.3 | 242.9 | 37.2 |
|  |  |  |  |  | Taiyuan | 559.0 | 728.7 | 169.7 | 30.4 |
| West | 47 037  (0.6) | 67 420  (1.0) | 20 382 | 43.3 | Guiyang | 193.7 | 511.1 | 317.4 | 163.8 |
|  |  |  |  |  | Chengdu | 665.1 | 1 572.9 | 907.8 | 136.5 |
|  |  |  |  |  | Chongqing | 504.1 | 943.1 | 439.0 | 87.1 |
|  |  |  |  |  | Urumqi | 533.6 | 914.5 | 380.9 | 71.4 |
|  |  |  |  |  | Kunming | 485.0 | 825.7 | 340.7 | 70.3 |
|  |  |  |  |  | Lhasa | 101.8 | 172.8 | 71.0 | 69.7 |
|  |  |  |  |  | Yinchuan | 438.0 | 666.8 | 228.8 | 52.2 |
|  |  |  |  |  | Lanzhou | 324.2 | 468.4 | 144.2 | 44.5 |
|  |  |  |  |  | Hohhot | 670.4 | 901.1 | 230.7 | 34.4 |
|  |  |  |  |  | Xining | 222.8 | 297.5 | 74.7 | 33.5 |
|  |  |  |  |  | Xi'an | 951.1 | 1 176.1 | 224.9 | 23.6 |
|  |  |  |  |  | Nanning | 1 011.5 | 1 070.0 | 58.5 | 5.8 |

The values in brackets list in second and third columns are the proportional cover of developed land. The city in our results included 32 provincial capital cities and Shenzhen which is one of fastest growing cities in China.

**Table S2. The contribution of urban expansion to land cover change for the whole nation, and the six urban megaregions.** Urban expansion was the dominant driver of farmland loss at the nation scale, but was the dominant driver for loss of forested land, farmland, and wetland in many of the urban megaregions.

|  | Forest | Grass | Farmland | Wetland | Other |
| --- | --- | --- | --- | --- | --- |
| Country | 15.0 | 10.3 | 43.2 | 11.4 | 9.3 |
| BTH | 14.2 | 14.4 | 50.2 | 29.0 | 23.9 |
| YRD | 61.5 | 16.2 | 65.7 | 73.3 | 33.5 |
| PRD | 57.4 | 5.4 | 70.5 | 40.4 | 14.2 |
| WH | 33.1 | 41.2 | 76.5 | 16.4 | 26.0 |
| CZT | 71.3 | 33.9 | 82.5 | 57.7 | 49.5 |
| CY | 47.8 | 29.4 | 43.7 | 33.0 | 10.7 |

The value in each column is the ratio of the land that converted to developed land to the total area of the land loss (%). For example, the first value in the forest column represents the ratio of the forest converted to developed land to the total area of the forest loss.

**Table S3. Area and proportional cover of the EVI trends.**

|  | Increase* | Increase | Decrease* | Decrease |
| --- | --- | --- | --- | --- |
| Country | 421.9×10^4^  (44.4) | 353.2×10^4^  (37.2) | 27.6×10^4^  (2.9) | 146.5×10^4^  (15.4) |
| Urban | 17 039  (23.8) | 14 424  (20.2) | 24 521  (34.3) | 15 490  (21.7) |
| Old urban | 10 216  (28.5) | 6 097  (23.0) | 5 235  (19.7) | 4 978  (18.8) |
| New urban | 6 823  (15.2) | 8 327  (18.5) | 19 286  (42.9) | 10 512  (23.4) |
| Non-urban | 4 201 423  (44.6) | 3 517 168  (37.3) | 251 817  (2.7) | 1 449 317  (15.4) |

* Coefficient is significant at the 0.05 level. The values outside of the brackets are the area of the four types of trends (km^2^), the values in the brackets are the proportional cover of the four types of trends (%).

**Table S4. Area and proportional cover of the LST trends.**

|  | Increase | Increase* | Decrease | Decrease* |
| --- | --- | --- | --- | --- |
| Country | 280×10^4^  (29.5) | 34.2×10^4^  (3.6) | 481.2×10^4^  (50.7) | 153.8×10^4^  (16.2) |
| Urban | 28 867  (42.2) | 12 283  (17.9) | 23 390  (34.2) | 3 907  (5.7) |
| Old urban | 9 406  (36.9) | 2 233  (8.7) | 11 719  (46.0) | 2 138  (8.4) |
| New urban | 19 461  (45.3) | 10 050  (23.4) | 11 671  (27.2) | 1 769  (4.1) |
| Non-urban | 2 760 961  (29.4) | 331 712  (3.5) | 4 770 387  (50.8) | 1 524 502  (16.3) |

* Coefficient is significant at the 0.05 level. The values outside of the brackets are the area of the four types of trends (km^2^), the values in the brackets are the proportional cover of the four types of trends (%).
